# Learning from medical errors

**DOI:** 10.1186/s42155-023-00406-6

**Published:** 2024-01-10

**Authors:** Joseph J. Gemmete

**Affiliations:** Department of Radiology, Neurology, Neurosurgery, and Otolaryngology, University of Michigan Hospitals, UH B1D 328, 1500 E Medical Center Drive, Ann Arbor, MI 48109 USA

## Introduction

In the complex and dynamic world of healthcare, medical errors, while regrettable, are an inevitable part of the learning process. The impact of such errors transcends beyond immediate patient care, resonating emotionally and professionally with healthcare providers. Yet, it is not the occurrence of these errors, but our response to them, that truly defines the trajectory of medical excellence. Through strategic approaches like education, simulation, debriefing, constructive feedback, peer support, and mentorship, the interventional radiologist has the potential to transform these setbacks into powerful learning opportunities, ensuring not only the enhancement of patient safety but also the professional growth of the interventional radiologist. There are intrinsic and extrinsic factors involved in the process of using medical errors for education purposes. In this manuscript we will discuss the following strategies of education, stimulation, debriefing, constructive feedback, peer support, and mentorship to learn from medical errors.

## Education

Education is crucial in the field of interventional radiology as it plays a fundamental role in preventing and addressing medical errors. Medical errors, which can range from diagnostic mistakes to procedural complications, and have profound implications for patient safety, quality of care, and healthcare costs. Adequate and continuous education of the interventional radiologist serves as a proactive measure to address these issues. Let's examine the relationship between education and medical errors:

### Foundational knowledge

Interventional radiologists require a solid educational foundation in radiology, anatomy, and physiology. This ensures that they are well-versed in the standard protocols, techniques, and best practices specific to their field. A strong foundational knowledge reduces the likelihood of basic mistakes during procedures [1].

### Skill development and competency

Hands-on training and simulation-based education are essential for interventional radiologists. These practical teaching methodologies help them develop and refine their procedural skills. Competency in performing complex interventions is crucial for minimizing errors that may arise from technical incompetence [2].

### Continuous Medical Education (CME)

Interventional radiology is a rapidly evolving field with continuous advancements in technology and techniques. CME programs are essential to keep interventional radiologists updated with the latest guidelines, equipment, and findings. Staying current with best practices is vital to providing safe and effective care [3].

### Cultural competence

Interventional radiologists must be culturally competent to provide patient-centered care. Education in cultural competence ensures that they can offer interventions that respect the diverse backgrounds, beliefs, and values of their patients, reducing the risk of errors due to misunderstandings or misalignment [4].

### Teamwork and communication

Effective communication and collaboration within multidisciplinary healthcare teams are critical in interventional radiology. Educational programs that emphasize teamwork and communication help prevent errors resulting from communication breakdowns among healthcare professionals involved in a patient's care [5].

### Understanding and addressing errors

Similar to other healthcare fields, interventional radiology teams should engage in post-procedure debriefings, root cause analyses, and reflective practices. These educational tools encourage the team to learn from any procedural errors, understand their root causes, and develop strategies to prevent their recurrence in the future [6].

### Patient education

Educating patients about interventional radiology procedures, potential risks, and post-procedure care is essential for reducing errors. Informed patients can actively participate in their care, follow pre-procedure instructions accurately, and help identify any concerns or complications early on [7].

### Systematic reviews and research

Educational research within interventional radiology can identify common areas where errors occur and propose evidence-based interventions. This research contributes to the overall improvement of safety in the field [8].

### Technology and informatics

Education on the effective use of imaging technology, specialized equipment, and electronic health records (EHRs) is crucial for interventional radiologists. Proper training can reduce errors related to imaging interpretation, procedure documentation, and patient data management [9].

In conclusion, education is a fundamental component of interventional radiology practice and is instrumental in preventing and addressing medical errors. By ensuring that interventional radiologists are well-trained, up-to-date, and equipped with the right skills and knowledge, the field can significantly enhance patient safety during minimally invasive procedures.

## Simulation

Simulation in the context of medical training and education is a powerful tool for enhancing patient safety and reducing medical errors in the field of interventional radiology. Simulation-based education provides interventional radiologists with a controlled environment to practice clinical skills, make mistakes, learn from them, and refine their techniques without posing any risk to real patients. Here's a more detailed look at the relationship between simulation and medical errors:

### Skill acquisition and refinement

Interventional radiologists perform intricate and minimally invasive procedures, such as angioplasty and stent placement. Simulations provide a controlled environment for these specialists to practice these procedures repeatedly, refine their techniques, and build muscle memory, thereby reducing the risk of technical errors during actual interventions [10].

### Team training

Many interventional radiology procedures involve a multidisciplinary team, including radiologic technologists, nurses, and anesthesiologists. Simulation scenarios can help improve teamwork and communication among team members, ensuring that everyone understands their roles and responsibilities. Effective teamwork reduces the likelihood of errors during procedures [11].

### Decision-making and critical thinking

Simulation-based scenarios challenge interventional radiologists to make rapid and well-informed decisions. These scenarios can mimic real-life situations where unexpected complications or anatomical variations occur, requiring quick thinking and adaptation. Practicing in a simulated environment enhances their ability to make critical decisions during actual procedures, ultimately reducing diagnostic and therapeutic errors [12, 13].

### Exposure to rare but critical events

Interventional radiologists may encounter rare and life-threatening complications during procedures, such as vascular perforations or contrast reactions. Simulations can expose them to these uncommon events, helping them develop the skills and confidence to manage such situations effectively and minimize potential errors [14, 15].

### Feedback and debriefing

Debriefing sessions following simulation scenarios are vital in interventional radiology training. They allow participants to review their performance, identify any errors or suboptimal techniques, and discuss strategies for improvement. This reflective process helps interventional radiologists learn from their mistakes and apply corrective actions in real clinical settings [16, 17].

### Reducing patient harm

The ability to practice and refine interventional radiology skills in a simulated environment reduces the risk of errors that could harm patients during actual procedures. By honing their skills and decision-making abilities in simulations, interventional radiologists enhance patient safety in clinical practice [18, 19].

In summary, simulation-based training in interventional radiology is a powerful approach to improving patient safety and reducing medical errors. It provides interventional radiologists with the opportunity to develop and enhance their technical skills, teamwork, decision-making abilities, and preparedness for rare but critical events. Through simulation, interventional radiologists can better serve their patients and minimize the risks associated with complex minimally invasive procedures.

## Debriefing

Debriefing is an essential component in the medical field, particularly when addressing medical errors. It offers the interventional radiologist an opportunity to reflect on their actions, decisions, and outcomes in a structured and supportive environment. Debriefing provides a constructive avenue for understanding the causes of errors, learning from them, and identifying strategies to prevent future occurrences. Here’s how debriefing relates to medical errors:

### Identification and acknowledgment

Debriefing sessions in interventional radiology create a safe space for team members to openly acknowledge any errors or unexpected outcomes that occurred during a procedure. This candid acknowledgment is the first step in addressing and rectifying medical errors, fostering a culture of transparency and accountability [20].

### Analyzing the root causes

In interventional radiology, where complex procedures are performed, errors can have multifaceted causes. Debriefings allow the healthcare team to delve deeper into the reasons behind the error, examining both immediate factors (e.g., procedural steps) and systemic factors (e.g., equipment, protocols). This thorough analysis helps identify the root causes and contributes to the development of targeted solutions [21].

### Promotion of open communication

Effective debriefing in interventional radiology encourages team members, including radiologists, technologists, and nurses, to share their perspectives and experiences. Open communication ensures that all team members have a comprehensive understanding of the event and its contributing factors, facilitating a more thorough analysis [22].

### Development of corrective actions

Once the causes of errors are understood through debriefing, the interventional radiology team can collaboratively develop corrective actions. These actions may involve revising protocols, enhancing communication strategies, implementing additional safety checks, or adjusting procedural techniques. The goal is to prevent similar errors from occurring in future procedures [23–25].

### Enhancing learning and professional growth

Debriefing sessions in interventional radiology serve as valuable learning opportunities. Team members can reflect on their performance, share insights, and collectively improve their skills, knowledge, and behaviors. This continuous learning process contributes to reducing the likelihood of errors and enhancing patient safety [26].

### Emotional support and coping

Medical errors in interventional radiology can have emotional consequences for healthcare professionals involved, including radiologists, nurses, and technologists. Debriefing provides a supportive environment where individuals can express their feelings, share their experiences, and receive emotional support from their colleagues. This support is essential for addressing the emotional impact of errors and promoting well-being among team members [27–29].

In summary, debriefing is an essential component of addressing medical errors in interventional radiology. It facilitates error identification, root cause analysis, open communication, corrective action development, continuous learning, and emotional support. By embracing debriefing as a structured and supportive practice, interventional radiology teams can improve patient safety, enhance the quality of care, and ensure the well-being of healthcare professionals involved in complex procedures.

## Constructive feedback

Constructive feedback plays a pivotal role in the context of medical errors. It provides an avenue for promoting understanding, encouraging learning, and facilitating improvements in clinical practice. Constructive feedback focuses on guiding the interventional radiologist toward optimal patient care by analyzing mistakes in a non-punitive manner and suggesting actionable solutions. Let's delve into the significance of constructive feedback concerning medical errors:

### Error recognition and accountability

In interventional radiology, where precise procedures are performed, constructive feedback helps healthcare professionals recognize errors and take ownership of their actions. It creates an environment where professionals can acknowledge mistakes without fear of punitive measures, promoting a culture of transparency and accountability [30].

### Facilitation of continuous learning

Constructive feedback is a catalyst for continuous learning in interventional radiology. It allows radiologists, technologists, and other team members to analyze errors, understand their root causes, and acquire new knowledge and skills to improve future patient care. Learning from mistakes is a cornerstone of professional growth [31].

### Promotion of open dialogue

Open communication is vital in interventional radiology to ensure the safety of patients. Constructive feedback encourages team members to openly discuss errors, share perspectives, and collaborate on solutions. This open dialogue is crucial for addressing errors comprehensively and preventing their recurrence [32].

### Reduction of future errors

Through constructive feedback, healthcare professionals can identify specific areas where errors occurred and work on targeted improvements. This proactive approach helps reduce the likelihood of similar errors happening in future interventional radiology procedures [33].

### Encouragement of reflection

Feedback prompts self-reflection among interventional radiology professionals. It allows them to critically assess their actions, decision-making processes, and procedural techniques. This reflection is essential for identifying areas where improvement is needed [34].

### Strengthening of clinical competence

Constructive feedback serves as a tool to strengthen clinical competence in interventional radiology. By highlighting areas of strength and offering guidance on areas of weakness, it enables professionals to enhance their skills and knowledge [35].

### Building of resilience and coping mechanisms

Medical errors in interventional radiology can be emotionally challenging. Constructive feedback provides a supportive environment where healthcare professionals can cope with the emotional impact of errors. This support contributes to building resilience and the ability to bounce back from adverse events [36].

In summary, constructive feedback in interventional radiology is integral to error recognition, learning, and continuous improvement. It fosters an environment where errors are seen as opportunities for growth and where patient safety remains paramount. Through constructive feedback, interventional radiology teams can enhance their skills, reduce the risk of errors, and ultimately provide the best possible care to their patients.

## Peer support

Peer support in the context of medical errors is a critical component in ensuring the well-being of interventional radiologists and fostering a culture of continuous learning and patient safety. When a medical error occurs, the interventional radiologist involved can experience profound emotional distress, often termed the "second victim" phenomenon. Peer support programs can offer understanding, empathy, and guidance during these challenging times. Let's explore the relationship between peer support and medical errors:

### Mitigating the "Second Victim" phenomenon

Medical errors in interventional radiology can have a significant emotional impact on healthcare professionals, including radiologists, technologists, and nurses. The "second victim" phenomenon is particularly relevant in this context. Peer support programs provide a safe space for these professionals to share their experiences, emotions, and challenges, helping them cope with feelings of guilt, shame, and anxiety that may arise after an error [6].

### Promotion of open dialogue

Peer support programs within interventional radiology create a culture of open and non-judgmental communication. Healthcare professionals can freely discuss errors they have been involved in, ensuring that they do not feel isolated or hesitant to seek help or share their experiences. This open dialogue is crucial for understanding the circumstances surrounding errors and learning from them [37].

### Facilitating learning and growth

Peer discussions about medical errors offer valuable opportunities for interventional radiology professionals to gain insights, reflect on their actions, and identify areas for improvement. By learning from each other's experiences, they can collectively work to prevent the recurrence of similar mistakes and enhance patient safety [38].

### Enhancing resilience

Peer support fosters a sense of camaraderie and solidarity among interventional radiology colleagues. This support network can bolster resilience and help professionals navigate the challenges of their practice, ultimately enabling them to bounce back more robustly after adverse events [39].

### Cultural shift towards patient safety

Peer support programs contribute to a cultural shift within interventional radiology, emphasizing patient safety over blame. By encouraging open discussions about errors and their underlying causes, these programs promote a collective commitment to improving healthcare delivery and minimizing the risk of future errors [40].

### Reintegration into clinical practice

Following a significant medical error, some interventional radiology professionals may feel apprehensive about returning to clinical duties. Peer support can play a crucial role in the reintegration process, ensuring that individuals receive the necessary support, guidance, and feedback to regain their confidence and competence in clinical practice [41].

In conclusion, peer support programs in interventional radiology are essential for addressing the emotional and professional challenges that arise in the aftermath of medical errors. By providing understanding, empathy, and a platform for shared learning, these programs contribute to the well-being of healthcare professionals and the continuous improvement of patient safety in the field of interventional radiology.

## Mentorship

Mentorship in the field of interventional radiology plays a significant role in shaping the approach, understanding, and management of medical errors. Through guidance, expertise, and support, mentors help their mentees navigate the complex world of healthcare, including the inevitable challenges associated with medical mistakes. Here's an exploration of the interplay between mentorship and medical errors:

### Education and prevention

Experienced IR mentors possess a wealth of knowledge and expertise in performing complex procedures. They can share their own experiences, including any past errors or near-misses, with their mentees. This sharing of insights and strategies helps mentees develop a deep understanding of potential pitfalls, enhancing error prevention efforts [42–44].

### A safe space for reflection

In interventional radiology, where precision and patient safety are paramount, encountering a medical error can be emotionally challenging for a young practitioner. Mentorship provides a safe and confidential space for the mentee to discuss the error, reflect upon it, and gain perspective without fear of judgment. This reflective process is essential for personal and professional growth [45, 46].

### Building resilience

Medical errors can have a lasting impact on the emotional well-being of IR practitioners. Mentors draw from their own experiences to help mentees develop coping mechanisms and emotional resilience. They offer guidance on how to navigate the emotional aftermath of errors, ensuring that mentees can bounce back and continue providing high-quality care [47].

### Navigating systemic challenges

Healthcare systems can sometimes contribute to errors in interventional radiology. Experienced mentors are well-versed in the intricacies of these systems and can guide mentees in recognizing and addressing systemic factors that may lead to errors. Together, they can work toward systemic improvements that enhance patient safety [48].

### Promoting a culture of accountability and learning

Effective mentorship in interventional radiology instills a culture where medical errors are seen as opportunities for learning and growth, not just as individual failures. Mentors emphasize the importance of taking responsibility for errors and actively engaging in continuous improvement efforts to enhance patient safety [49, 50].

### Emotional and professional support

Encountering a medical error can be a defining moment in an IR practitioner's career. Mentors provide both emotional and professional support, helping mentees navigate feelings of guilt, self-doubt, or anxiety. This support ensures that mentees can continue their career progression and remain committed to patient safety [51, 52].

In conclusion, mentorship in interventional radiology plays a crucial role in addressing and learning from medical errors. Through guidance, reflection, and unwavering support, mentors assist mentees in transforming challenging experiences into opportunities for growth, learning, and systemic improvement. The mentor–mentee relationship fosters a culture within IR that prioritizes patient safety, continuous learning, and the personal and professional development of IR practitioners.

## Conclusion

Learning from medical errors is not merely a process but a commitment to continuous improvement in healthcare. Implantation of these strategies can provide a safer environment for patients and interventional radiologists and accurately identify safety challenges while implementing a plan through education, training, and teamwork rather than a culture of blame, fear, and punishment to negate medical errors. By leveraging strategies such as education, simulation, debriefing, constructive feedback, peer support, and mentorship, we can instill a culture where mistakes are no longer stigmatized but are seen as catalysts for growth. Such an environment promotes transparency, collaboration, and resilience, ensuring that each error becomes a bridge towards a more competent and compassionate healthcare system. Embracing these strategies not only safeguards our patients but also nurtures and supports the interventional radiologist dedicated to their care.

## Data Availability

Not applicable.
